# Exploring the Influence of Oral and Gut Microbiota on Ulcerative Mucositis: A Pilot Cohort Study

**DOI:** 10.1111/odi.15246

**Published:** 2025-01-06

**Authors:** Valentin Bartha, Sébastien Boutin, Dorothée L. Schüßler, Anna Felten, Shila Fazeli, Florentina Kosely, Thomas Luft, Diana Wolff, Cornelia Frese, Kyrill Schoilew

**Affiliations:** ^1^ Department for Conservative Dentistry, School of Dental Medicine University Heidelberg Heidelberg Germany; ^2^ Department of Infectious Diseases and Microbiology University of Lübeck and University Medical Center Schleswig‐Holstein Campus Lübeck Lübeck Germany; ^3^ Airway Research Center North (ARCN) German Center for Lung Research (DZL) Lübeck Germany; ^4^ Clinic for Cardiology, Pneumology, Angiology Und Internal Intensive Care Medicine Ludwigshafen Hospital Ludwigshafen Germany; ^5^ Internal Medicine V: Hematology, Oncology Und Rheumatology Heidelberg University Hospital Heidelberg Germany; ^6^ Institute for Oral Health Interlaken Switzerland

**Keywords:** allogeneic stem cell transplantation, microbiome, mucositis, ulcerations

## Abstract

**Aim:**

Comparing oral and gut microbiome profiles between patients with and without ulcerative mucositis during allogeneic stem cell transplantation (aSCT).

**Materials and Methods:**

Specimens from oral mucosa, saliva, and stool were collected pre‐(T0) and post‐ (T0 +28d ± 14d) aSCT (T1). Microbiome structure differences were analyzed by 16S‐rRNA‐gene sequencing, and associations to patients' clinical characteristics were investigated.

**Results:**

Ten of 25 included patients developed ulcerations. The α‐diversity decreased between T0 and T1, independent of ulcerations. PERMANOVA revealed differences in beta diversity between T1 stool samples from patients with and without ulcerations. At T1, saliva samples of patients with ulcerations showed an increase of *Mycoplasma salvarius, while commensals decreased in saliva and mucosal swabs*. The gut microbiome of both groups showed an overabundance of *Enterococcus* spp., associated with inflammatory conditions. Salival α‐diversity of older and overweight patients decreased slower, whereas in mucosal swabs mucositis or impaired renal function was associated with a higher decline. Female gender and history of periodontitis were associated with increased stool microbiome changes, while self‐reported probiotics intake was related to reduced changes.

**Conclusions:**

Ulcerations appeared in 40% of the patients. Distinct microbial changes, including increased abundance of Mycoplasma salivarius in saliva and decreased abundance of commensals, marked those with ulcerations.

**Trial Registration:**

The study was registered in the German Register for Clinical Studies (DRKS00032882)

## 1 Introduction

The pathogenesis of oral mucositis is characterized by a complex interplay between the epithelial and subepithelial connective tissue compartments (Sonis 2004). The process includes the expression of proinflammatory cytokines and culminates in tissue damage through a combination of apoptosis and tissue necrosis (Sonis 2011) accompanied by a decrease of neutrophils (Hong et al. 2019) and strong microbial alterations (Bruno et al. 2023). Especially the microbial role has been the subject of research for several years (Bruno et al. 2023). Perturbations to the microbial homeostasis, promoted by various intrinsic and extrinsic factors, induce microbial dysbiosis, possibly favoring the proliferation of potentially pathogenic microorganisms (Lamont, Koo, and Hajishengallis 2018; Marsh, Head, and Devine 2015). Dysbiosis has been described as a microbial community state that contributes to the etiology of a related disease. It is characterized by compositional and functional alteration of the microbiome and can be driven by factors such as inflammation, xenobiotics, diet, and environmental factors (Levy et al. 2017). In terms of bacterial diversity, dysbiosis is commonly characterized by a decrease of diversity compared to the microbial diversity under healthy conditions and by an increased dominance of pathological microbiota, characterized by a permanent loss of some microorganisms, with others outgrowing and persisting (Petersen and Round 2014). However, in contrast to this definition, specifically in the context of periodontal diseases, dysbiosis has been described as characterized by a strong increase of microbial diversity—still accompanied by a shift of the composition towards pathogens (Mombelli 2018). Cancer therapies, such as allogeneic stem cell transplantations (aSCT), are typically accompanied by cytotoxic treatments and broad‐spectrum antibiotics that exert a substantial impact on the biofilm's ecological stability, promoting dysbiosis (Kesavelu and Jog 2023; Khan et al. 2019; Laheij and de Soet 2014). It is known that the effects of antibiotics on microbial balance within the gut increase tissue permeability, allowing pathogens and microbial products to enter deeper layers and the bloodstream, consecutively affecting distant tissues, including oral niches (Yuan et al. 2023). Such effects of antibiotics can be further enhanced by unfavorable dietary habits (Lee et al. 2020). Moreover, pre‐existing antibiotic resistances might contribute to the definition of the microbiome structure that establishes during such cancer therapies. It is known that both the gut and the oral microbiome can be a reservoir for antibiotic resistance genes (Anderson et al. 2023; McInnes et al. 2020).

Although several studies investigated the microbiome during different phases of oral mucositis (Bruno et al. 2022; Hong et al. 2019; Hou et al. 2018; Laheij et al. 2019; Reyes‐Gibby et al. 2020; Vesty et al. 2020; Zhu et al. 2017), still, its exact role in this process remains not fully understood. Especially the bidirectional microbe‐host relationship complicates answering the question regarding cause and effect (Bruno et al. 2023). Two studies indicated associations between the microbiome composition at baseline of cancer therapy and the mucositis severity (Bruno et al. 2022; Zhu et al. 2017). Moreover, the composition of the gut microbiome before treatment seems to be linked to the severity of oral mucositis (Al‐Qadami et al. 2023). The composition of the gut microbiome and its metabolism can affect the muco‐epithelial barrier and enhance inflammatory responses (Chakaroun, Massier, and Kovacs 2020; Veziant et al. 2021), while the oral cavity can act as a reservoir for pathogens that affect the gut microbiome (Atarashi et al. 2017; Bao et al. 2022). In this context, animal studies demonstrated that *Porphyromonas gingivalis* and *Klebsiella spp*. can induce dysbiosis of the gut microbiome (Arimatsu et al. 2014; Atarashi et al. 2017; Nakajima et al. 2015). Moreover, oral relative abundance of *Porphyromonas gingivalis* before the conditioning regimen was correlated with more oral mucositis grades (Bruno et al. 2022).

Due to the lack of studies investigating oral and gut microbiomes simultaneously, this longitudinal pilot study aimed to compare the oral and gut microbiomes in patients undergoing aSCT. It was primarily hypothesized that the severity of mucositis and the occurrence of ulcers are associated with differences in microbiota diversity parameters and microbiota abundances in these patients. Secondarily, possible correlations with patients' oral, clinical, and habitual characteristics before aSCT should be investigated.

## 2 Materials and Methods

### 2.1 Study Design

This study was designed as a longitudinal, prospective pilot cohort study, adhering to the STROBE Statement for cohort studies (see Table S1). The study was conducted at the University of Heidelberg, University Hospital, Department of Conservative Dentistry, and Clinic for Internal Medicine V: Hematology, Oncology, and Rheumatology.

The study design was approved by the University of Heidelberg Ethics Committee, with the Stem Cell Transplantation Section establishing a biobank in 2002, aligned with allogeneic stem cell transplantations. For this particular project, the sample collection expanded upon the existing biomaterial bank, necessitating an amendment submission to the local Ethics Committee (Amendment S‐120/2002). The study was registered in the German Register for Clinical Studies (DRKS00032882). Comprehensive verbal and written information about the study was provided to all patients.

### 2.2 Inclusion Criteria

- Patients scheduled to undergo allogeneic stem cell transplantation (aSCT) due to hematological diseases.
- Age ≥ 18 years.
- Patients able to give written informed consent for participation in the study.

### 2.3 Exclusion Criteria

- Patients who did not consent to providing samples at both time points (T0 and T1).

### 2.4 Patient Questionnaire, Oral Examination, and Sample Collection

Patients underwent a dental history questionnaire. Saliva, mucosal biofilm, and stool samples were collected before (T0) and 28 days (±14 days) post‐allogeneic stem cell transplantation (T1) using standardized and validated techniques (OMNIgene ORAL OMR‐110, OM‐505/OMNIgene GUT OM‐200, DNA Genotek, Canada). The OMNIgene collection kits are validated and CE‐marked devices that allow for the collection, stabilization, storage, and transportation of saliva, mucosal swabs, and gut samples at ambient temperature—ensuring the microbial profile of the sample accurately represents the in vivo profile. Samples were collected following the standardized and validated instructions of the manufacturer. Mucosal swab samples were obtained from the participants' planum buccale. Continuous monitoring of oral mucous membranes was a routine practice, with oral mucositis classification according to Seegenschmiedt (2006). Dental status assessments were conducted one to two times a week by four calibrated dentists (K.S., A.F., S.F., D.S.). The calibration procedure was conducted as long as an interrater reliability of at least 95% regarding the plaque index and the grading of mucositis according to WHO classification (World Health Organization 1979) was achieved. Dental parameters, including the record of the number of decayed (D), missing (M), and filled (F) teeth (T) using the DMF‐T Index (Roland et al. 1994) and estimating the amount of dental plaque using a plaque index (Silness and Löe 1964), were recorded using magnifying glasses and lights directly at the patient's bedside to avoid additional patient transport. Relevant covariates such as oral hygiene, dietary habits, fluoridation, educational level, self‐reported history of periodontitis, intake of probiotics, and smoking status were determined through a standardized questionnaire. The conditioning regimen, supportive drugs, and the dosage in the case of total body irradiation were taken from the patients' charts. Due to the heightened immunosuppressed state post‐allogeneic stem cell transplantation, further periodontal examinations were renounced in order not to increase the risk of bacteremia. Consequently, the periodontal health status was approximated through the questionnaire (see Table S2). Additionally, self‐reported mouth dryness was assessed using the four point Likert scale of the European Organization for Research and Treatment of Cancer Quality of Life Questionnaire Head and Neck 35.

Oral mucositis was diagnosed according to the WHO classification (World Health Organization 1979) with Grade 0: No mucositis; Grade 1: Soreness/erythema; Grade 2: Erythema, ulcers, patient can swallow solid food; Grade 3: Ulcers with extensive erythema, patient cannot swallow solid food; and Grade 4: Severe ulcers requiring parenteral or enteral nutrition.

### 2.5 Baseline Examination (T0)

Upon admission, patients completed a series of questionnaires addressing sociodemographic and psychosocial parameters, including the EORTC QLQ‐C30 and EORTC QLQ‐OH15, along with questions specific to oral health (Appendix [Link], [Link], [Link], [Link]). Patients were instructed to collect a stool sample using the OM‐200 kit, label it with their name and date, and bring the packaged stool sample and completed questionnaires in the T0 envelope to the hospital.

At the hospital, healthcare personnel collected mucosal swabs from the buccal mucosa and vestibule (left and right sides) using the OMR‐110 kit and unstimulated saliva samples with the OM‐505 kit. These samples were labeled with the patient's name and date and enclosed in the T0 envelope.

During the inpatient stay, patients completed daily questionnaires on mucositis symptoms when possible.

### 2.6 Dental Examination

A calibrated dental examiner (DS, AF) conducted an oral health assessment. The examiner collected the T0 envelope and samples and provided the patients with T1 envelopes for subsequent sample and questionnaire collection.

### 2.7 Follow‐Up Collection (T1), 28 ± 14 Days Post‐Transplantation

Patients again completed psychosocial questionnaires (EORTC QLQ‐C30 and EORTC QLQ‐OH15) and provided a stool sample using the OM‐200 kit, labeled with name and date, and placed in the T1 envelope with the completed questionnaires.

Healthcare personnel collected a second set of mucosal swabs (OMR‐110 kit, buccal mucosa and vestibule, both sides) and unstimulated saliva samples (OM‐505 kit), labeled with patient identifiers, and stored them in the T1 envelope.

The dental examiner retrieved the T1 envelope and associated samples at the time of discharge.

#### 2.7.1 DNA Extraction

All samples were stored at −80°C in the S1 laboratory of the Department of Conservative Dentistry at the Dental Clinic until DNA extraction. Following the manufacturer's guidelines for sample collection kits, mucosal swab DNA extraction utilized the Epicentre MasterPure Complete DNA and RNA Purification Kit, while stool sample DNA extraction employed the QIAGEN QIAamp PowerFecal DNA Kit. Negative controls, using sterile water, were incorporated to mitigate potential contamination.

### 2.8 Library Preparation for Next Generation Sequencing (NGS)

DNA was amplified using universal bacterial primers flanking the V4 region (primer sequence: 515F; 5′‐*GTGCCAGCMGCCGCGGTAA‐3*′, 806R; 5′‐GGACTACHVHHHTWTCTAAT*‐3*′.). Each primer was tagged with a spacer of 2–4 nucleotides and the sequencing adapter. Polymerase chain reactions (PCRs) were performed in 25‐μL volumes composed of Q5 High‐Fidelity 1× Master Mix (New England Biolabs GmbH, Germany), 25 pmol of each primer, and 2 μL of DNA. Thermal cycling (Primus 25, Peqlab Biotechnologie GmbH, Germany, or FlexCycler2, Analytik Jena AG, Germany) conditions were: first denaturation at 94°C for 3 min, 30 amplification cycles (94°C for 45 s, 50°C for 1 min, and 72°C for 1 min 30 s), and a final extended cycle at 72°C for 10 min. PCR products were purified using magnetic beads MagSi‐NGSprep Plus (Steinbrenner Laborsystem GmbH, Wiesenbach, Germany) and processed through a second step amplification with primers targeting the sequencing adapter region and tagged with individual barcodes and the Illumina i5/i7 sequences. Each barcode is unique and differs by at least 3 nucleotides from all other barcodes to assign sequences to the samples. PCR products were purified with magnetic beads MagSi‐NGSprep Plus (Steinbrenner Laborsystem GmBH, Wiesenbach, Germany) and quantified using the Quant‐iT PicoGreen dsDNA Assay Kit (Thermo Fisher Scientific, Karlsruhe, Germany). Samples were pooled as an equimolar mix and sequenced on a Miseq system (Illumina Inc., San Diego, CA, USA) (600 cycles). PCR was also performed with negative controls from the extraction and with sterile water to exclude contamination from the extraction kit and the PCR master mix. Internal control was performed by amplifying a mock community sample containing genomic DNA from 20 bacterial strains in equimolar (even) ribosomal RNA operon counts (HMD‐782D, BEI Resources, ATCC, Manassas, VA, USA).

### 2.9 Bioinformatic Analysis

The raw sequence data was processed using Dada2 to produce amplicon sequence variants (ASVs) with the following parameters: no ambiguities (N) allowed, one error per read allowed, and truncation of the reads at the first position with a quality score < 2. Reads were merged as contigs and checked for chimeras with the default parameters. The read counts of each ASV were then used to create an ASV table, and the sequences were classified using the Silva database v138.1. Those data were merged in a phyloseq object in R, adding the metadata.

Decontamination of the data was done using the R package decontam. The bacterial community was characterized by calculating α‐diversity (Shannon index) and β‐diversity (Morisita‐Horn distance). Statistical differences between groups regarding the α‐diversity were evaluated using a linear mixed effects model taking into account patient identity, including time points, ulceration status, age, gender, and antibiotic usage (number of antibiotics used) as co‐variables. PERMANOVA was used to compare the difference between groups in the β‐diversity (Morisita‐Horn distance) using the same co‐variables. Differential abundance between groups was evaluated using MaAsLin2 with the significant co‐variables from the PERMANOVA as fixed effects and the patient ID as a random effect. Exploratory associations between self‐reported questionnaire results (e.g., behavioral and clinical parameters) and microbiome diversity metrics were assessed using Spearman rank correlation.

### 2.10 Statistical Methods

The sample size for this exploratory analysis was not based on formal power calculations but was determined by the availability of participants and resources during the recruitment period, in line with the pilot nature of the study. It was limited by the beginning of the COVID‐19 pandemic in March 2020, one year after the start of recruitment. Given the novel nature of microbiome analyses in the context of aSCT at the time of study planning (2016–2017) and the lack of data to perform precise power calculations, the cohort size was designed to provide preliminary data and inform future, more comprehensive studies. Clinical data and data from the questionnaire were documented in Excel (Microsoft Corp., Redmond, USA) and subsequently transferred to JMP15 (SAS Institute Inc., Cary, USA) for statistical analysis. Variables were tested for normal distribution using the Shapiro–Wilk test. Given the non‐normal distribution of most continuous variables, median values and 25% and 75% quartiles were calculated. Intergroup differences between patients with and without ulcerations were assessed using the Mann–Whitney *U* Test for continuous variables and the Pearson‐Chi‐square‐Test for categorical variables. The significance level (alpha level) was set at 0.05.

## 3 Results

**FIGURE 1:**
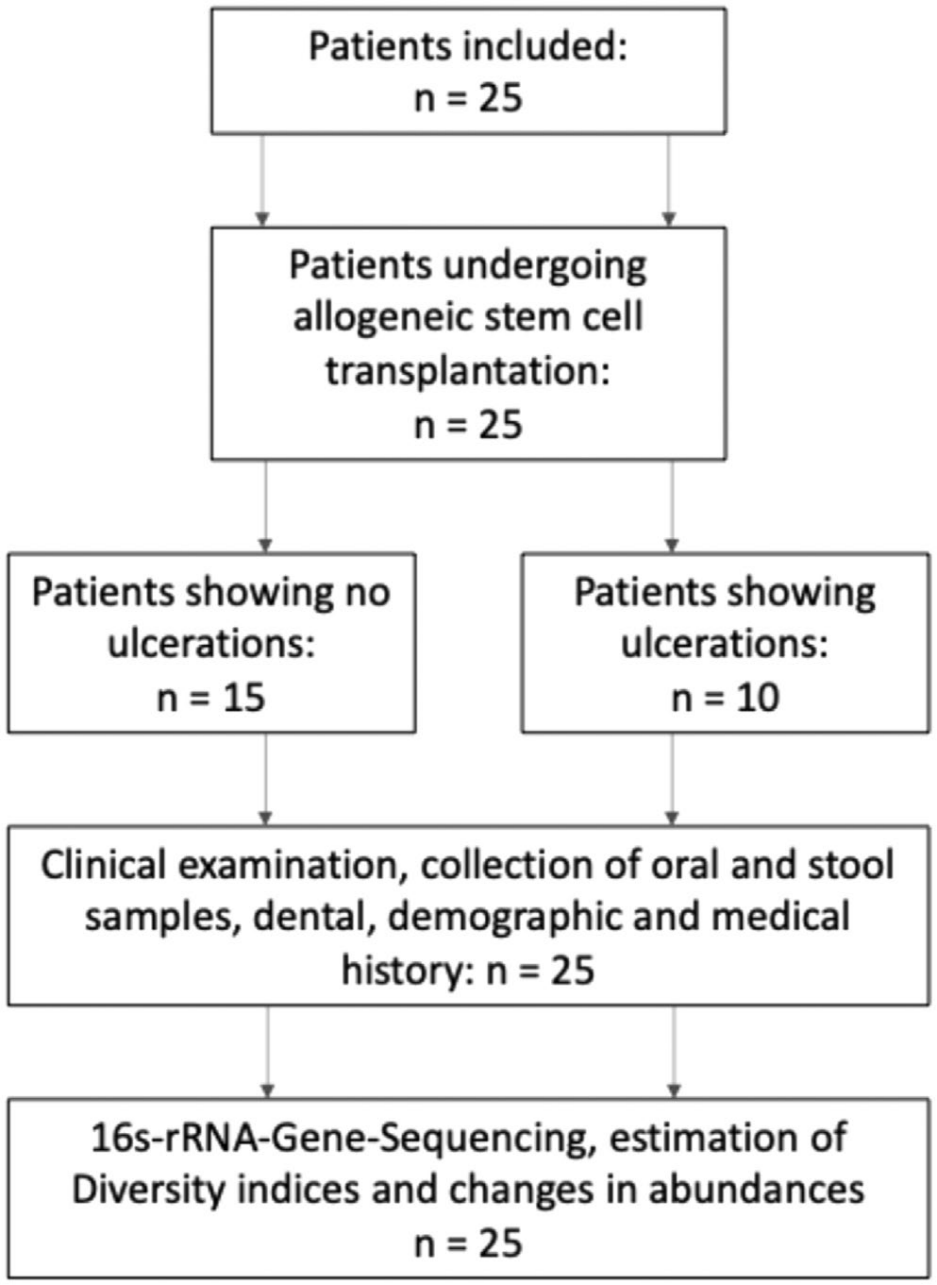
Study flow chart.

In total, 29 patients could initially be recruited; four patients declined participation during T0, not willing to participate. An analysis was conducted on the data of 25 patients undergoing allogeneic stem cell transplantation, representing a cohort with a median age of 60 years and 32% female (Figure 1). Among the participants, seven exhibited mucositis grade 1 without ulcerations, eight showed no signs of mucositis, while 10 patients presented more severe mucositis with ulcerations. Among the latter group, four showed mucositis grade 2, four grade 3, and two grade 4. Due to the low number of participants in each group, for further analysis, the cohort was divided into two subgroups: patients without ulcerations (u−) and patients with ulcerations (u+). The median body weight of the overall cohort was 75 kg (IQR 63–92.8), with a BMI analysis revealing that 48% of all patients were classified as overweight, with no significant differences observed between the groups with or without ulcerations.

### 3.1 Conditioning Regimen, Supportive Drugs and Total Body Irradiation

Most patients received Fludrabine (*n* = 23) and Treosulfan (*n* = 7) in combination with various other conditioning agents, presented in Table S3. In total, 23 patients additionally received Anti‐Thymocyte globoline (ATG); three patients received chemotherapy (high‐dose cytarabine + mitoxantrone, *n* = 3, or Daunorubicin, *n* = 1). Moreover, the magnitude of patients received Cyclosporin A (*n* = 23) as an immunosuppressant drug—again, in many cases in combination with ATG (*n* = 13) and Methotrexat (*n* = 16). There were no statistically significant differences between the groups. Other immunosuppressant drugs were CellCept, mycophenolate, Sirolimus, Tacrolimus, and different Glucocorticoids.

All patients received Cotrim Forte as a broad‐spectrum antibiotic as part of their standard care. Additionally, they received Rifaximin (*n* = 13), Ciprofloxacin (*n* = 4), Tazobactam (*n* = 6), Amoxicillin with Clavulanic acid (*n* = 5), Meropenem (*n* = 3), Amikacin (*n* = 1), and Ceftazidime/Avibactam (*n* = 1). Again, there was no statistically significant difference in the distribution of the antibiotic regimens between patients with and without ulcerations (*p* = 0.315). Morevover, an antimycotic and an antiviral medication were given to all patients as supportive medication. 14 patients received supportive vitamin D.

TBI was applied in 10 patients, thereof six with a dosage of 8 Gy, three with 6 Gy and one with 2 Gy. Table S3 provides an overview regarding the conditioning regimens and TBI.

### 3.2 Socioeconomic and Disease Data

Analysis of socioeconomic data revealed no significant differences in school or university degrees among the patient groups. The underlying diseases varied, with acute myeloid leukemia (AML) being the most common (*n* = 10). Among the patients with AML, five exhibited mucositis with ulcerations. Two patients with ulcers suffered from chronic lymphatic leukemia, two from follicular lymphoma, and one from myelofibrosis (Table 1).

**TABLE 1:** Characteristics of all patients and of patients with (u+) or without (u−) mucosal ulcerations and distribution of various diagnoses among patients with ulcerations (u+) and those without ulcerations.

	All	u−	u+	Intergroup *p*
*n*	25	15	10	
Age (range)	60 (24–75)	61 (24–69)	52.5 (35–75)	0.222^a^
Female *n* (%)	8 (32%)	5 (20%)	3 (12%)	0.861^b^
Weight kg (median, IQR)	75 (63–92.8)	73 (63–91)	82.5 (65.25–101.25)	0.617^a^
BMI (kg/m^2^; median, IQR)	24.5 (22.4–28.2)	23.9 (22,3. 27.3)	26.3 (22.8–30.8)	0.360^a^
BMI category *n* (%)				
Normal	13 (52)	9 (36)	4 (16)	0.327^b^
Over‐weight	12 (48)	6 (24)	6 (24)	
High school *n* (%)	6 (24)	4 (16)	2 (8)	0.485^b^
University degree *n* (%)	2 (8)	1 (4)	1 (4)	0.958^b^
Patient history of periodontitis *n* (%)	8 (32)	5 (20)	3 (12)	0.964^b^
Diagnosis *n* (%)				
ALL	1 (4)	1 (4)	0	0.373^b^
AML	10 (40)	5 (20)	5 (20)	
CLL	3 (12)	1 (4)	2 (8)	
DLBCL	1 (4)	1 (4)	0 (0)	
FL	2 (8)	0 (0)	2 (8)	
HSCT	1 (4)	1 (4)	0 (0)	
MDS	2 (8)	2 (8)	0 (0)	
MM	1 (4)	1 (4)	0 (0)	
Myelofibrosis	2 (8)	1 (4)	1 (4)	
T‐PLL	2 (8)	2 (8)	0 (0)	

Abbreviations: ALL, acute lymphatic leukemia; AML, acute myeloic leukemia; BMI, body mass index; CLL, chronic lymphatic leukemia; DLBCL, diffuse large B‐cell lymphoma; FL, follicular lymphoma; HSCD, homozygote sickle cell disease; MDS, myelodysblastic disease; MM, multiple myeloma; T‐PLL, T‐prolymphocytic leukemia; u, ulceration.

^a^ Mann–Whitney‐*U*‐Test.

^b^ Pearson‐Chi‐Square‐Test.

### 3.3 Clinical and Behavioral Data

Comparisons between the groups with ulcerations (u+) and without ulcerations (u−) showed no statistically significant differences in DMF‐T values. The median DMF‐T for patients without ulcerations was 14 (12–26), compared to 13.25 (6.75–18.25) in the group with ulcerations. Similarly, the median Plaque Index was 0.63 in the u− group versus 0.64 in the u+ group (Figure S1). Furthermore, there were no statistically significant differences in the self‐reported mouth dryness and periodontal health status between the groups.

Additional assessments, including self‐reported sugar consumption, intake of probiotics, other dietary habits, and domestic dental hygiene procedures, also demonstrated no significant differences between the two groups (data not shown).

### 3.4 Overall Sequencing Data

The mean number of total reads per sample was 11,545 ± 3905 reads. Each sample reached the plateau on a rarefaction curve, indicating sufficient coverage (Figure S2). The replication of the Mock community showed low to no variation in the microbial composition as well as no sign of contamination (Table S4). Negative controls were sequenced, and 9 RSVs were found belonging to the genera *Cutibacterium*, Comamonadaceae unclassified, *Bradyrhizobium*, *Pseudoxanthomonas*, *Marmoricola*, and *Phaselicystis* as well as mitochondrial DNA. Those ASVs were excluded from the dataset using the package decontam.

### 3.5 Associations Between aSCT and Ulceration Status With the Salivary Microbiota

**FIGURE 2:**
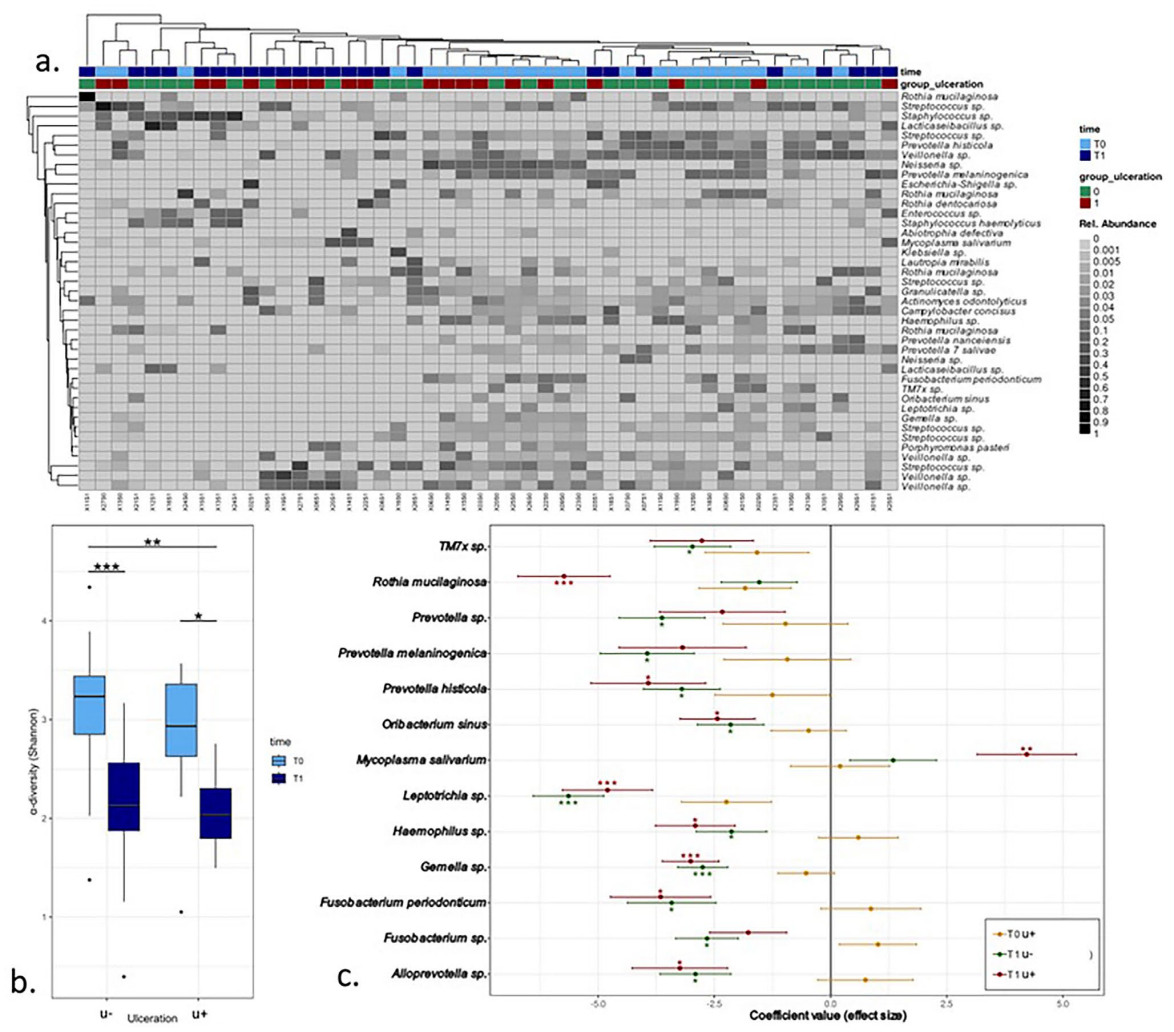
(a) Heatmap, representing the most prevalent RSVs in saliva (average relative abundance > 0.005). (b) Alpha‐diversity (Shannon index) for the saliva samples, illustrating the changes before (T0) and after (T1) stem cell transplantation, distinguishing between those without ulcers (u−) and those with ulcers (u+). (c) MaAsLin2 analyzing RSV abundances in saliva for groups and timepoints with group u− at T0 as reference. *p < 0.05; **p < 0.01; ***p < 0.001.

In the saliva, the most abundant taxa belonged to the genera *Streptococcus*, *Veillonella*, *Neisseria*, and *Prevotella* (Figure 2a). Associated with time between T0 and T1, we observed a significant change in beta‐diversity in the overall cohort (PERMANOVA, *R* ^2^ = 0.10, adjusted *p*‐value = 0.001), along with marginally non‐significant changes in patients with ulcerations (PERMANOVA, *R* ^2^ = 0.03, adjusted *p*‐value = 0.055). There was a significant influence of gender (PERMANOVA, *R* ^2^ = 0.03, adjusted *p*‐value = 0.022) and the number of antibiotics (PERMANOVA, *R* ^2^ = 0.04, adjusted *p*‐value = 0.01) taken in the context of aSCT. Principal coordinate analysis of the Morsita‐horn distances for all samples and timepoints is presented in Figure S3.

Samples taken after transplantation showed a higher dominance by a single taxon, and it was validated by a significant decrease of α‐diversity in both groups after transplantation (Tukey's range test: u−: adjusted *p*‐value < 0.001; Figures 2b and S4). We did not observe a significant difference in the α‐diversity in patients with ulcerations compared to no ulcerations at the single time points. In regards to taxa enrichment, we observed no significant differences in RSV abundances between the groups at T0. There was an increased relative abundance in *Mycoplasma salivarium* and a lower abundance of *Rothia mucilaginosa, Prevotella histicola, Oribacterium sinus, Leptotrichia sp., Haemopilus sp., Gemella sp., Fusobacterium periodonticum*, and *Alloprevotella sp*. compared to samples from patients without ulcerations at T1. Similarly, we observed a decreased relative abundance of *TM7X sp., Prevotella sp., Oribacterium sinus, Leptotrichia sp., Haemopilus sp., Gemella sp., Fusobacterium periodonticum, Fusobacterium sp*., and *Alloprevotella sp*. (Figure 2c).

### 3.6 Associations Between aSCT and Ulceration Status With the Oral Mucosal Microbiota

**FIGURE 3:**
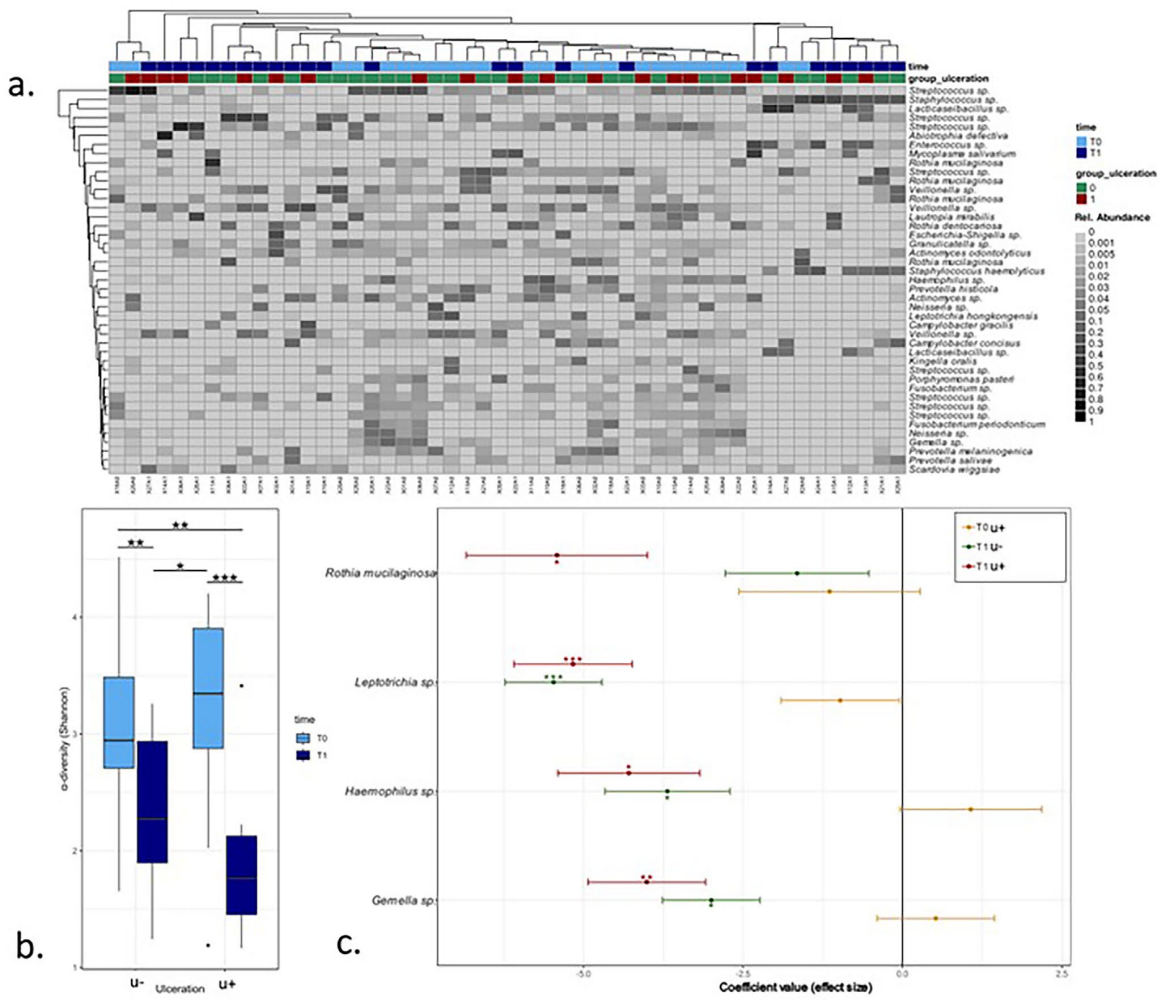
(a) Heatmap, representing the most prevalent RSV in the oral mucosa (average relative abundance > 0.005). (b) Alpha‐diversity (Shannon index) of the microbiota in the oral mucosa, illustrating the changes before (T0) and after (T1) stem cell transplantation, distinguishing between those without ulcers (u−) and those with ulcers (u+). (c) MaAsLin2 analyzing RSV abundances in saliva for groups and timepoints with group u− at T0 as reference. *p < 0.05; **p < 0.01; ***p < 0.001.

In the oral mucosa, the microbiome composition was closely related to the saliva's microbiome (PERMANOVA, *R* ^2^ = 0.03, adjusted *p*‐value = 0.018; Figure S2); the most abundant taxa also belonged to the genera *Streptococcus*, *Veillonella*, *Neisseria*, and *Prevotella* (Figure 3a). Similar to saliva, associated with time between T0 and T1, we observed a significant change in beta‐diversity in the overall cohort (PERMANOVA, *R* ^2^ = 0.07, adjusted *p*‐value = 0.001), along with a significant influence of gender (PERMANOVA, *R* ^2^ = 0.04, adjusted *p*‐value = 0.014) and the number of antibiotics (PERMANOVA, *R* ^2^ = 0.05, adjusted *p*‐value = 0.001). Samples from oral mucosa taken after transplantation (T1) showed a significant decrease of α‐diversity in both groups after transplantation without any impact of ulceration (Tukey's range test: u−: adjusted *p*‐value < 0.001; u+: adjusted *p*‐value < 0.001; Figure 3b). Figure S4 demonstrates the changes in dominance. As for the saliva, we observed an overall lower abundance in *Haemophilus sp*. and *Gemella sp*. in patients without ulcerations at T1 and additionally of *Rothia mucilaginosa* and *Leptotrichia sp*. in patients with ulcerations at T1. However, in contrast to the saliva microbiome, we did not observe any increase in relative abundance at T1 in any groups (Figure 3c).

### 3.7 Associations Between aSCT and Ulceration Status With the Stool Microbiota

**FIGURE 4:**
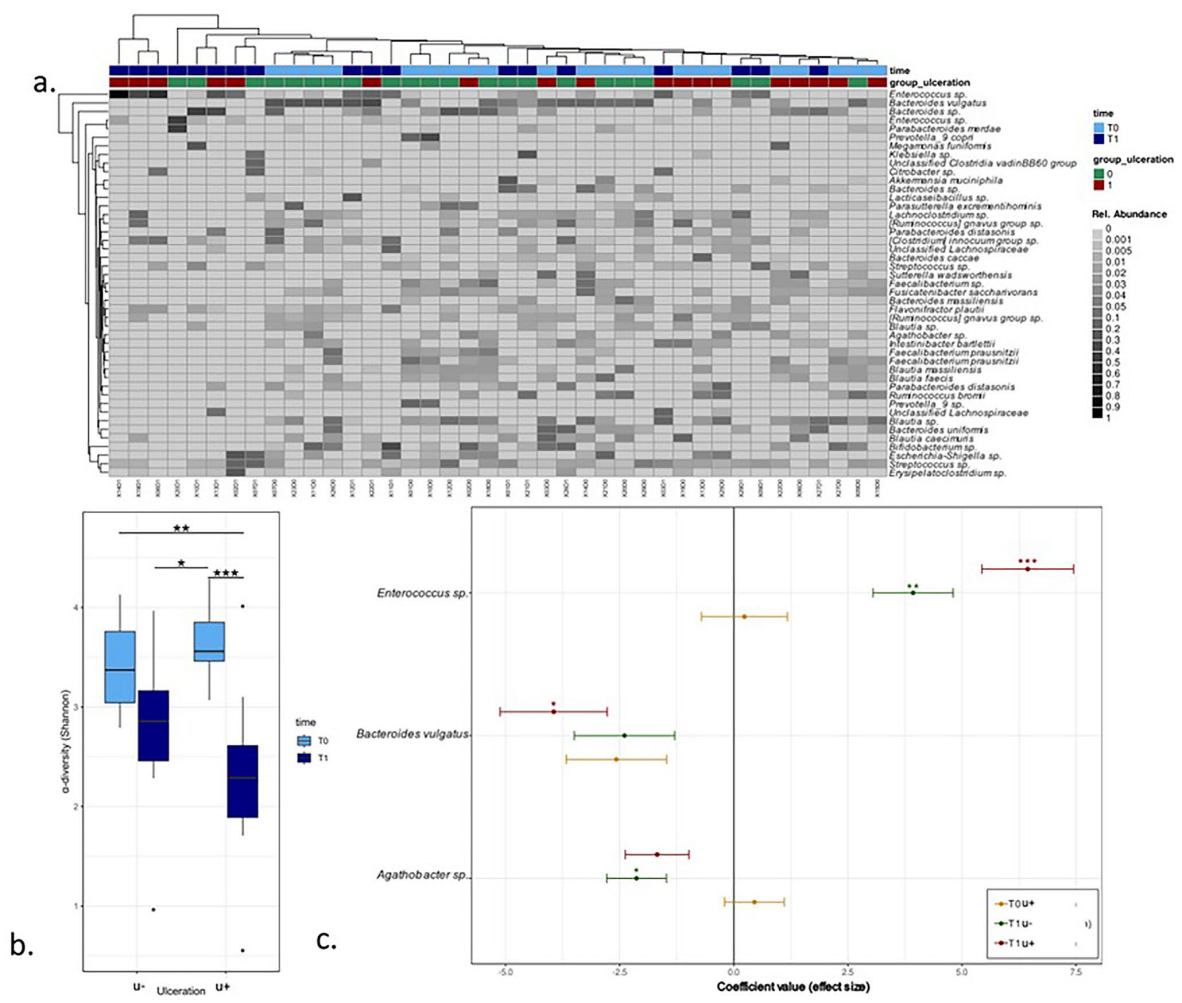
(a) Heatmap, representing the most prevalent RSV in the stool (average relative abundance > 0.005). (b) Alpha‐diversity (Shannon index) of the microbiota in the oral mucosa, illustrating the changes before (T0) and after (T1) stem cell transplantation, distinguishing between those without ulcers (u−) and those with ulcers (u+). (c) MaAsLin2 analyzing RSV abundances in saliva for groups and timepoints with group u− at T0 as reference. *p < 0.05; **p < 0.01; ***p < 0.001.

The stool microbiota was significantly different from the two other compartments (stool vs. saliva PERMANOVA, *R* ^2^ = 0.14, adjusted *p*‐value = 0.003; Stool vs. oral mucosa PERMANOVA, *R* ^2^ = 0.12, adjusted *p*‐value = 0.003; Figure S3). The most abundant taxa belonged to the genera *Bacteroides*, *Enterococcus*, *Bifidobacterium*, and *Blautia* (Figure 4a). There was a significant effect of time (PERMANOVA, *R* ^2^ = 0.10, adjusted *p*‐value = 0.001), occurrence of ulceration (PERMANOVA, *R* ^2^ = 0.06, adjusted *p*‐value = 0.003), and the interaction of time and ulceration on β‐diversity (PERMANOVA, *R* ^2^ = 0.03, adjusted *p*‐value = 0.047), indicating a shift in the β‐diversity at T1 between patients with and without ulceration. In contrast to saliva and mucosal swab samples, there was no effect of sex and the number of antibiotics on the beta diversity. The α‐diversity was decreased after aSCT in both groups without a difference between samples of patients with and without ulcerations (Tukey's range test: u−: adjusted *p*‐value = 0.048; u+: adjusted *p*‐value < 0.001; Figure 4b). Figure S4 demonstrates the changes in dominance. We observed an increased abundance of *Enterococcus* species in both groups at T1. Moreover, in patients with ulcerations, *Bacteroides vulgaris* was decreased at T1, while *Agathobacter sp*. were decreased in patients without ulceration (Figure 4c).

### 3.8 Behavioral and Clinical Parameters and Their Associations With the Microbiota of the Saliva, Mucosa, and Stool

As we observed similar trends between patients with and without ulceration we analyzed, sample‐type‐wise, potential confounding factors that might impact the decrease in α‐diversity and major changes in the β‐diversity during the aSCT. Regarding the decrease in α‐diversity in the saliva samples, we observed a significant association with age (Estimate: 0.029, *p*‐value 0.033), body mass index (Estimate: 0.7157, *p*‐value 0.027), and DMF‐T score (Estimate: 0.04092, *p*‐value 0.046), indicating that older and overweight patients showed a slower decline in α‐diversity. The body mass index also had a relation to the β‐diversity of the saliva's microbiome (Estimate: −0.060, *p*‐value = 0.029), indicating that overweight patients had a less pronounced change in their microbial structure after aSCT.

Regarding the microbiome of the mucosa, we observed a statistical effect of mucositis (Estimate: −0.232, *p*‐value = 0.041) and impaired renal function (Estimate: −1.06, *p*‐value = 0.044) indicating that patients with mucositis or impaired renal function could have a higher decline in α‐diversity.

The decline in α‐diversity of the gut microbiome was associated with differences in gender, with females showing a higher decline (Estimate: 1.179, *p* = 0.006) and with self‐reported history of periodontitis, where patients with periodontitis showed a higher decline as well (Estimate: −1.0941, *p* = 0.027). The β‐diversity in the stool was affected by the number of missing teeth (Estimate: −0.007, *p* = 0.011) and the DMF_T score (Estimate: −0.006, *p* = 0.043), indicating that poor oral health status correlated with higher change in the gut microbiota after aSCT. The usage of extra oral health accessories such as flossing devices and tongue brushes was also associated with higher changes in the β‐diversity (Estimate: 0.095, *p* = 0.047). Finally, the self‐reported usage of probiotics in the time before aSCT reduced the change in ß‐diversity of the stool microbiome in the period before and after aSCT (Estimate: −0.132, *p* = 0.027).

## 4 Discussion

In this cohort of patients undergoing allogeneic stem cell transplantation, we found a statistically significant decline in α‐diversity across all three investigated compartments, though this did not appear to be significantly associated with the development of ulcerations. Moreover, shifts in overall bacterial composition, specifically in beta‐diversity, were found in the saliva and stool microbiomes of patients with ulcerations and in the mucosal microbiome of patients without ulcerations. However, in the mucosal samples of patients with ulcerations, the changes of the microbiome structure during an SCT approached the threshold of significance (*p* = 0.055) but still was statistically non‐significant. The β‐diversity of saliva and mucosal swab samples were significantly influenced by the number of different broad‐spectrum antibiotics administered during the aSCT period, as well as by gender. Additionally, specific bacterial taxa showed significant changes in abundance before and after aSCT.

Concordant with these findings, numerous studies have reported a cancer therapy‐related decrease in α‐diversity and variations in ß‐diversity among different mucositis severity grades within the oral mucosal microbiome (Bruno et al. 2022; Hong et al. 2019; Hou et al. 2018; Reyes‐Gibby et al. 2020; Vesty et al. 2020; Zhu et al. 2017) and within the saliva microbiome (Hong et al. 2019; Laheij et al. 2019; Shouval et al. 2020; Vesty et al. 2020).

Interestingly, we found no differences in α‐diversity between ulcer and non‐ulcer samples in any investigated compartment. During aSCT, the decline of α‐diversity is promoted by the numerous accompanying drugs, including combinations of broad‐spectrum antibiotics, antimycotics, immunosuppressives, and dietary changes (Kesavelu and Jog 2023; Khan et al. 2019; Laheij et al. 2019; Lee et al. 2020; Zhang, Ju, and Zuo 2018).

This indicates that such microbiome changes towards a less diverse and more dysbiotic microbiome occur globally across multiple environments, independent of the development of ulcerations. Antibiotic regimens and a gender dependency on such microbial shifts were described in previous studies (Gebri et al. 2020; Shono et al. 2016; Shouval et al. 2020).

A few factors from patients´ clinical data and history were identified to be correlated to the observed aSCT‐related microbiome changes. These included impaired renal function, owerweight, and age. As these findings are a result of an unadjusted statistical model in a cohort with a low number of participants, they should not be overinterpreted. However, some similar findings were reported previously. Regarding impaired renal function, it was reported that kidney diseases can be related to certain increased saliva metabolites, which are able to affect bacterial activity and inflammation (Dziarski and Gupta 2006; Kovalčíková et al. 2020; Tavares et al. 2022). Moreover, an effect of different age groups on the bacterial profiles of supragingival plaque was shown (Kazarina et al. 2023). Higher body weights can be related to more gingival inflammation due to circulations of proinflammatory cytokines produced by visceral fat and their local appearance in the oral cavity (García‐Sánchez et al. 2020). The role of these observations in the context of aSCT should be further investigated in trials with larger cohorts.

At the level of specific microbiota, at T0 there were no differences in RSV abundances between the groups. At T1, the u + saliva samples showed an increased abundance of *Mycoplasma salivarium, Mycoplasma salivarium* is associated with oral carcinomas, periodontitis, oral lichen planus, and arthritis (Henrich et al. 2014; Johnson, Bruckner, and Collins 2007; Mizuki et al. 2017; Sugiyama et al. 2016). Additionally, the T1 oral samples from patients with ulcerations exhibited a lower relative abundance of commensal, gram‐positive species like *Rothia mucilaginosa* (Baker et al. 2021). A relative depletion of commensals could be one factor that weakens the integrity of the microbiome resilience. At the same time, the relative increase of pathobionts like *Mycoplasma salivarium* might drive inflammation and contribute to the formation of ulcers. In contrast, the T1 samples of patients without ulcerations showed a lower abundance of certain *Prevotella* and *Fusobacterium* species, both able to act as pathobionts and linked to inflammation in the oral cavity (Han and Wang 2013; Könönen et al. 2022).

Regarding the significant reduction in α‐diversity of the stool microbiome, without differences between ulcerations and non‐ulcerations, along with a significant difference in ß‐diversity between both the time points and the groups, these results are supported by previous research (Al‐Qadami et al. 2023). We observed limited differences in RSV abundances between patients who developed ulcerations and those who did not. At T1, Enterococcus species, known to be related to antibiotic‐induced dysbiosis and weakening the mucosal barrier function (Almeida‐Santos et al. 2021), were more abundant in both groups. Similarly, in both groups, commensal species were less abundant; hence, there was no significant difference. This absence of substantial distinctions in stool microbiota might give a hint that local factors within the oral environment may play a more direct role in the pathogenesis of ulcerations post‐aSCT. At the same time it underscores the need to further explore compartment‐specific microbial responses to aSCT.

The analysis of clinical, medical history, and questionnaire data in the context of the stool microbiome has the same limitations as mentioned earlier in the context of the oral microbiome. The calculations suggest an association of self‐reported history of periodontitis with a lower α‐diversity of the stool samples. Moreover, a self‐reported intake of probiotics was related to reduced changes in ß‐diversity of the stool microbiome during aSCT. Although these findings should be interpreted very cautiously, they shall be reflected with some previously conducted studies. A few studies reported an impact of a dysbiotic oral microbiome on systemic diseases, including dysbiosis of the gut microbiome (Bao et al. 2022; Kleinstein, Nelson, and Freire 2020; Qian et al. 2022). Both, the bloodstream and swallowing of saliva acted as mechanisms of bacterial transmission from the oral cavity to the gut (Bao et al. 2022; Kleinstein, Nelson, and Freire 2020; Qian et al. 2022). A potential preventive role of probiotics in the prevention and mitigation of oral mucositis when applied before cancer therapy was reported within a systematic review of Liu, Wu, and Huang (2022). However, our data have the strong limitation of the self‐reported nature and furthermore do not provide information regarding the probiotics which were taken or how long they were taken. Moreover, we did not find any difference between u+ and u− and it has to be mentioned that the use of probiotics after aSCT is currently not recommended (Andermann, Rezvani, and Bhatt 2016).

In summary, based on our data and concordant with previous research, it could be cautiously interpreted that a dysbiotic microbiome, enriched with pathogens like *Mycoplasma salvarium*, could be one factor contributing to the severity of mucositis. It is currently unknown if the pre‐existent antibiotic resistome further contributes to the development of a pronounced dysbiosis that could support ulceration development. There might be an influence, and this could be a topic for further research. Moreover, we were unable to assess whether salivary flow rate varied among participants. We observed a self‐reported increase in mouth dryness from a questionnaire with no differences between the ulcers and non‐ulcers. Existing literature has highlighted that both aSCT‐related medications and TBI are associated with reduced salivary gland function, which can lead to reduced salivary flow rate that potentially contributes to altering of the oral microbiome (Chaushu et al. 1995; Mauramo et al. 2017; van Leeuwen et al. 2019). Future studies could include this topic. Additionally, they could focus on stabilizing oral and gut microbiomes before sSCT, especially the investigation of beneficial diets (Myles 2014; Rinninella et al. 2019; Zhang, Ju, and Zuo 2018), probiotics (Shu et al. 2020), and the role of preexisting chronic oral diseases that are related to dysbiosis, such as periodontal diseases.

Several limitations should be addressed: (1) The study has a low sample size, partly as a result of the beginning of the COVD‐19 pandemic, 1 year after the start of recruitment. (2) The study's findings may not be generalizable due to a lack of diversity in the patient cohort. Factors such as age, gender, and underlying health conditions should be more varied in future studies to get a broader generalizability of the results. A more diverse cohort could indicate how such variables might influence the microbiome's response to aSCT, as they generally affect immune function, microbiota composition, and overall health outcomes. Moreover, it would help to understand how different patient demographics and health statuses may contribute to microbiome alterations and the severity of mucositis more comprehensively. (3) The study's duration may not have been sufficient to observe long‐term microbiome changes and their impact on patient outcomes post‐therapy. Both an extended pretherapy period and an extended follow‐up period could provide more comprehensive insights. (4) A key limitation is the variation in follow‐up periods for sample collection (28 ± 14 days), potentially introducing confounding factors. Especially the timing of sample collection might have had an effect on the consistency of the presented microbiome changes, in particular affecting the microbiome shifts observed at T1. Future studies should aim for a more standardized follow‐up. Additionally, an extended follow‐up period would allow for investigate long‐term impacts of aSCT on the microbiome and patient outcomes. (5) No periodontal examination was conducted based on the immunosuppression of the participants. (6) Limited data on diet behavior pretherapy and after aSCT was accessed. Hence the effect of a possible malnutrition (high intake of sugar and industrially fermented carbohydrates, saturated fatty acids, trans fatty acids, low intake of dietary fiber, micronutrients, polyunsaturated fatty acids, and phytochemicals) could not be assessed. (7) Data regarding the history of periodontitis or intake of supplements like probiotics stem from a questionnaire. (8) Another limitation of the study is the missing analysis of body mass, in particular the inability to differentiate between lean and fat mass. This distinction is relevant, as the type of body mass—e.g., visceral fat—can have varying effects on metabolic processes and affect inflammation, which in turn may influence microbiome composition. Hence, future studies should aim to incorporate more detailed body composition assessments, such as distinguishing between lean and fat mass, aiming to provide a deeper understanding of the body composition's role on microbiome changes during aSCT.

Hence, in future studies, pretherapy examinations should include a diet assessment using a validated food frequency questionnaire or several 24 h diet protocols. Moreover, the nutrition post‐therapy should be adequately evaluated. Moreover, investigating the effects of intentional microbiome modulation, such as through prebiotics, probiotics, or dietary interventions, on patient outcomes would still be a promising area of research. Employing a multi‐omics approach, integrating metagenomics, metabolomics, and transcriptomics, could provide a more holistic understanding of the microbiome's role and its interaction with host biology.

Within the limitations of this study, it can be concluded that aSCT was associated with observable alterations in the microbial community as well as significant shifts in bacterial diversity and abundance. The microbial changes were limited to certain bacterial species. Ulcerations appeared in 40% of the patients. A higher relative abundance of *Mycoplasma salvarium* after aSCT, along with a decrease of commensal species, was observed in patients who later developed ulcerations, suggesting that this might contribute to the occurrence of more severe mucositis grades.

## Author Contributions

**Valentin Bartha:** writing – original draft, formal analysis, visualization, investigation, data curation. **Sébastien Boutin:** formal analysis, visualization, investigation, methodology. **Dorothée L. Schüßler:** investigation, data curation. **Anna Felten:** investigation, data curation. **Shila Fazeli:** investigation, data curation. **Florentina Kosely:** investigation, data curation. **Thomas Luft:** conceptualization, methodology, supervision, project administration. **Diana Wolff:** supervision, resources, writing – review and editing. **Cornelia Frese:** conceptualization, funding acquisition, resources, project administration. **Kyrill Schoilew:** conceptualization, investigation, funding acquisition, writing – review and editing, data curation.

## Ethics Statement

The study design was approved by the local ethics committee, with the Stem Cell Transplantation Section establishing a biomaterial bank in 2002, aligned with allogeneic stem cell transplantations. For this particular project, the sample collection expanded upon the existing biomaterial bank, necessitating an amendment submission to the local Ethics Committee (Amendment S‐120/2002).

## Consent

Informed consent was given from all participants included in this study.

## Conflicts of Interest

The authors declare no conflicts of interest.

## Supporting information


**Figure S1.** Boxplots depicting values for decayed, missing, and filled teeth (a) as well as for the plaque index (b), comparing the groups with (u+) and without (u‐) ulcerations.

**Figure S2.** Rarefaction curves for each sample included in the study. Samples are color‐coded based on the type of samples: Saliva (green), mucosal swab (orange), or stool (red).

**Figure S3.** Principal coordinate analysis of the Morsita‐horn distances regarding sample material and timepoints.

**Figure S4.** Changes in dominance within saliva, mucosal swab, and stool samples of patients with ulcerations (1) and without ulcerations (0).

**Table S1.** STROBE Statement‐checklist of items that should be included in reports of observational studies.

**Table S2.** Questionnaire participants had to fulfill regarding self‐reported periodontal health.

**Table S3.** Number of patients with Conditioning drugs, Total Body Irradiation (TBI) and other supportive medications.

**Table S4.** Reads counts for the mock community with 3 replicates.

**Appendix S1.** Fragebögen Teil 1–3.

**Appendix S2.** QLQ‐C30 German.

**Appendix S3.** OH15 German.

**Appendix S4.** Fragebögen Teil 4.

## Data Availability

The datasets generated and/or analyzed during the current study are available with the following link: https://www.ncbi.nlm.nih.gov/sra/PRJNA1175548.
