# Supplementary material for: Exploring the Influence of Oral and Gut Microbiota on Ulcerative Mucositis: A Pilot Cohort Study
Source: Oral Dis. 2025 Jan 6;31(6):1776–88. doi: 10.1111/odi.15246 (PMC12291438; doi:10.1111/odi.15246)
Supplement: Supplementary file 1 — Figure S1. Boxplots depicting values for decayed, missing, and filled teeth (a) as well as for the plaque index (b), comparing the groups with (u+) and without (u‐) ulcerations. [file ODI-31-1776-s006.docx]

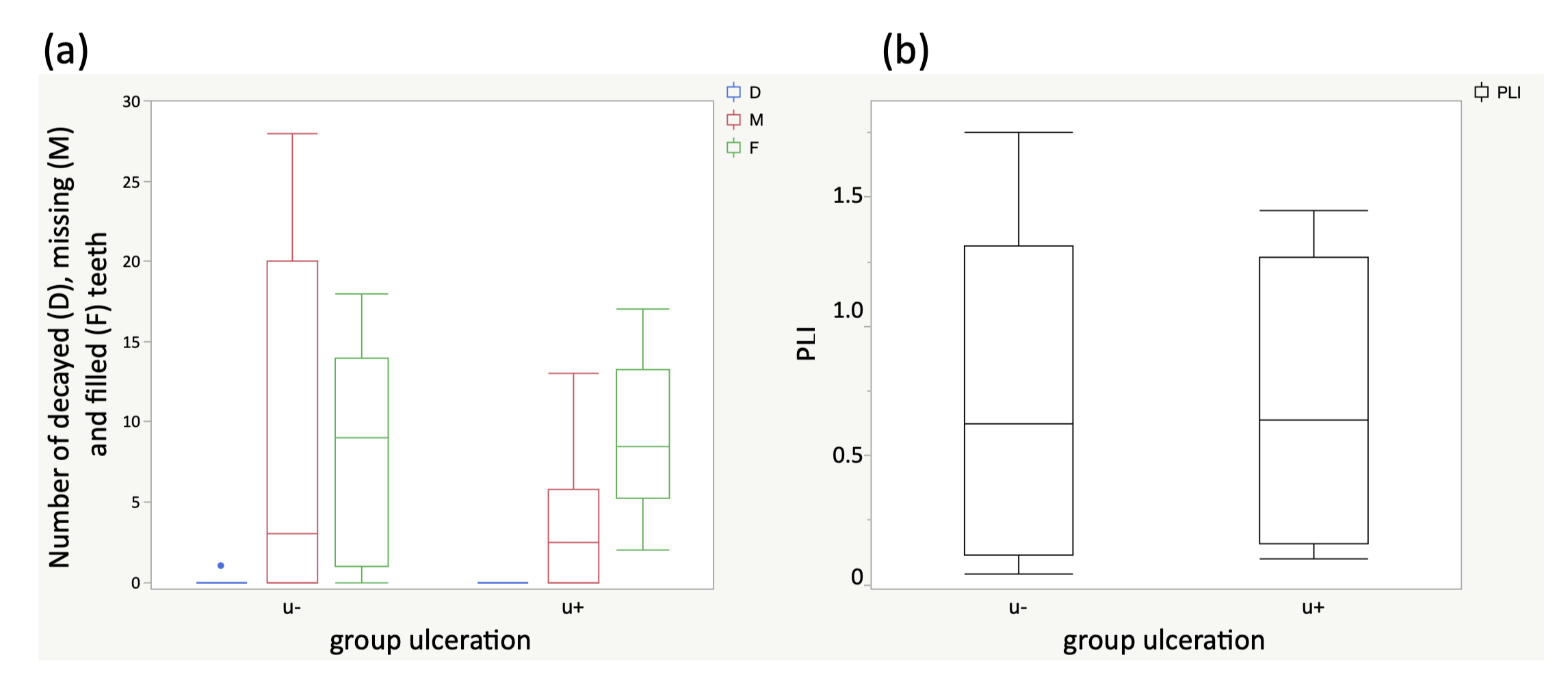


Supplementary Figure 1: Boxplots depicting values for decayed, missing, and filled teeth (a) as well as for the plaque index (b), comparing the groups with (u+) and without (u-) ulcerations.
