# Supplementary material for: Exploring the Influence of Oral and Gut Microbiota on Ulcerative Mucositis: A Pilot Cohort Study
Source: Oral Dis. 2025 Jan 6;31(6):1776–88. doi: 10.1111/odi.15246 (PMC12291438; doi:10.1111/odi.15246)

Supplementary figure 2: Rarefaction curves for each samples included in the study. Samples are color coded based on the type of samples : Saliva (green), mucosal swab (orange) or stool (red)


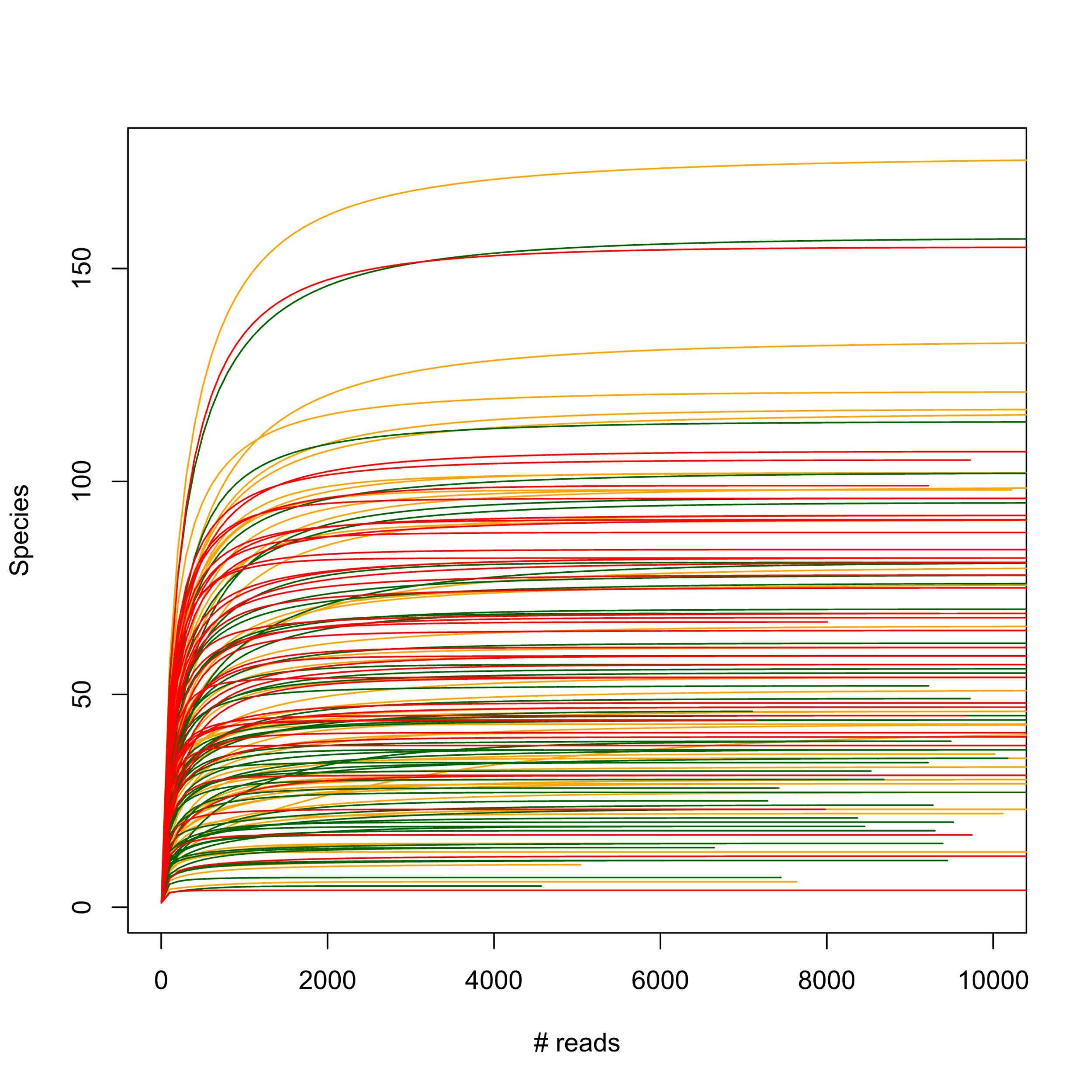

Supplement: Supplementary file 2 — Figure S2. Rarefaction curves for each sample included in the study. Samples are color‐coded based on the type of samples: Saliva (green), mucosal swab (orange), or stool (red). [file ODI-31-1776-s004.docx]
