# Supplementary material for: Exploring the Influence of Oral and Gut Microbiota on Ulcerative Mucositis: A Pilot Cohort Study
Source: Oral Dis. 2025 Jan 6;31(6):1776–88. doi: 10.1111/odi.15246 (PMC12291438; doi:10.1111/odi.15246)

Supplementary Figure 3: Principal coordinate analysis of the Morsita-horn distances regarding sample material and timepoints


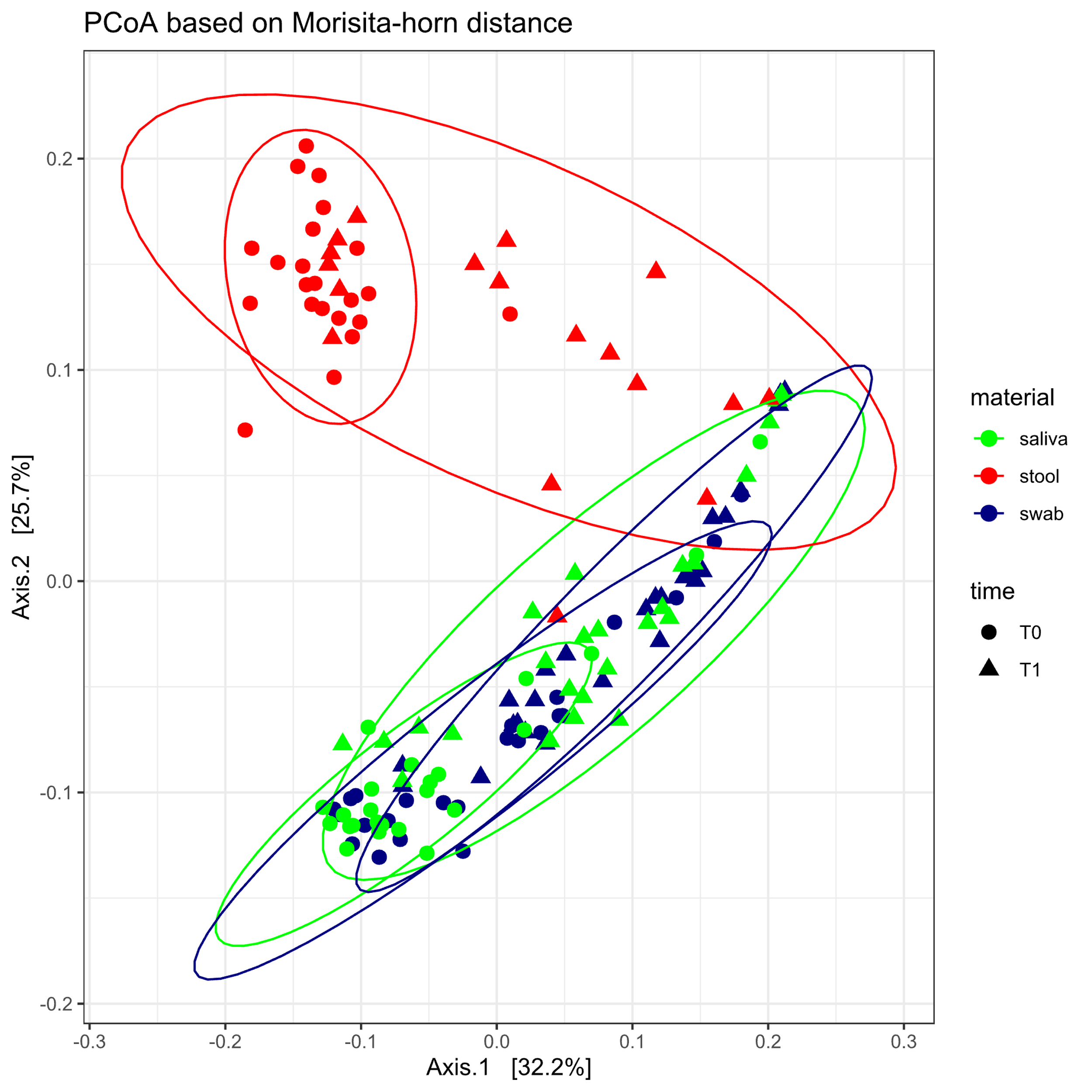

Supplement: Supplementary file 3 — Figure S3. Principal coordinate analysis of the Morsita‐horn distances regarding sample material and timepoints. [file ODI-31-1776-s002.docx]
