# Supplementary material for: Exploring the Influence of Oral and Gut Microbiota on Ulcerative Mucositis: A Pilot Cohort Study
Source: Oral Dis. 2025 Jan 6;31(6):1776–88. doi: 10.1111/odi.15246 (PMC12291438; doi:10.1111/odi.15246)

Supplementary Figure 4: Changes in dominance within saliva, mucosal swab and stool samples of patients with ulcerations (1) and without ulcerations (0)


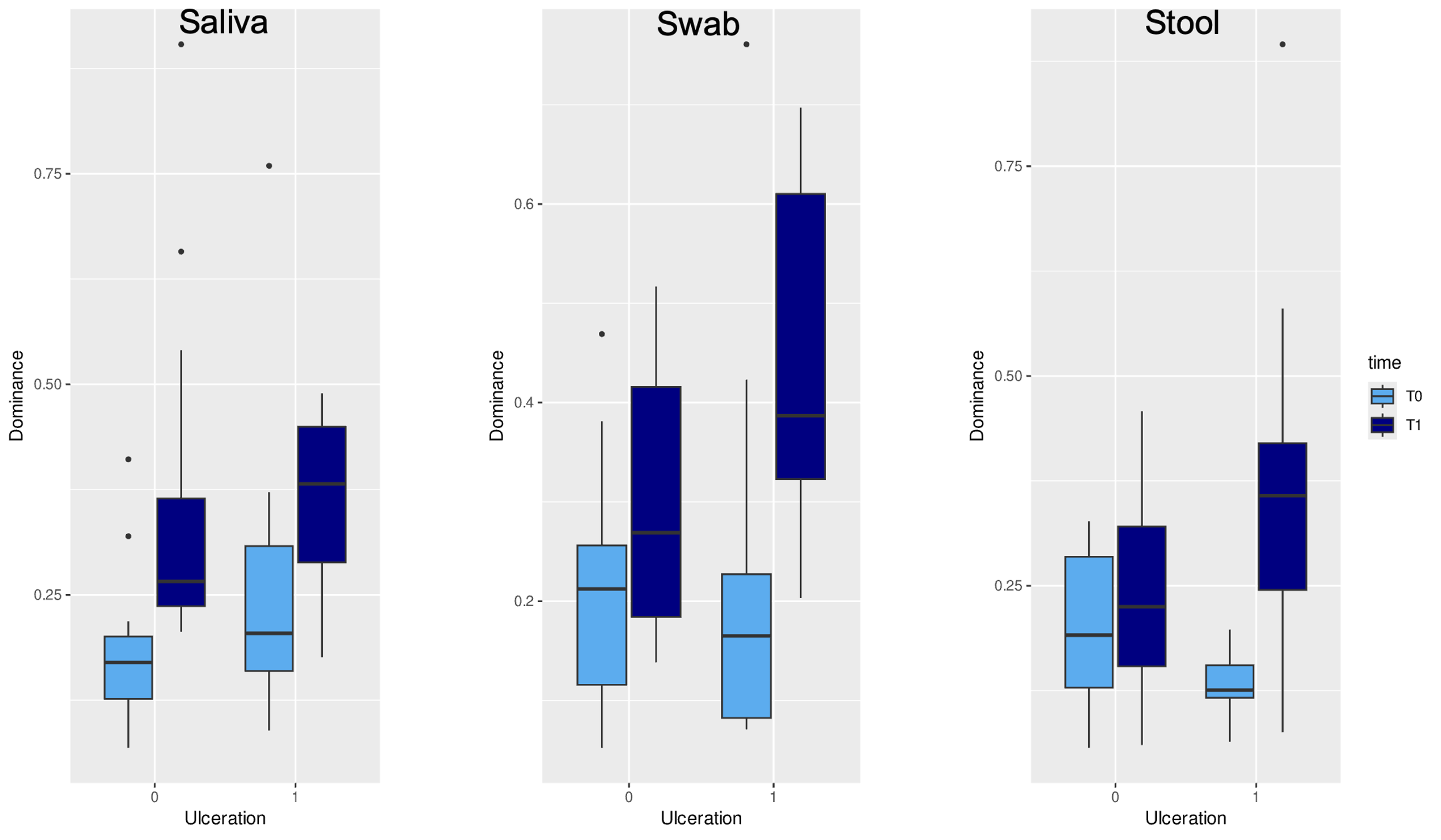

Supplement: Supplementary file 4 — Figure S4. Changes in dominance within saliva, mucosal swab, and stool samples of patients with ulcerations (1) and without ulcerations (0). [file ODI-31-1776-s008.docx]
