# Supplementary material for: Exploring the Influence of Oral and Gut Microbiota on Ulcerative Mucositis: A Pilot Cohort Study
Source: Oral Dis. 2025 Jan 6;31(6):1776–88. doi: 10.1111/odi.15246 (PMC12291438; doi:10.1111/odi.15246)
Supplement: Supplementary file 5 — Table S1. STROBE Statement‐checklist of items that should be included in reports of observational studies. [file ODI-31-1776-s007.docx]

STROBE Statement—checklist of items that should be included in reports of observational studies

|  | Item No. | Recommendation | Page  No. | Relevant text from manuscript |
| --- | --- | --- | --- | --- |
| **Title and abstract** | 1 | (*a*) Indicate the study’s design with a commonly used term in the title or the abstract | 1 | **Exploring the Influence of Oral and Gut Microbiota on Ulcerative Mucositis: a Pilot Cohort Study** |
|  |  | (*b*) Provide in the abstract an informative and balanced summary of what was done and what was found | 2 | See the abstract on page 3 |
| Introduction | | | |  |
| Background/rationale | 2 | Explain the scientific background and rationale for the investigation being reported | 4 | “Although several studies investigated the microbiome during different phases of oral mucositis (Bruno et al., 2022; Hong et al., 2019; Hou et al., 2018; Laheij et al., 2019; Reyes-Gibby et al., 2020; Vesty et al., 2020; Zhu et al., 2017), its exact role in this process remains not fully understood. Still, the microbiome before the start of cancer treatment is rarely investigated.”  And  “Due to the lack of studies investigating oral and gut microbiomes simultaneously,…” |
| Objectives | 3 | State specific objectives, including any prespecified hypotheses | 5 | …this longitudinal pilot study aimed to compare the oral and gut microbiomes in patients undergoing aSCT. It was primary hypothesized that the severity of mucositis and the occurrence of ulcers are associated with differences in microbiota diversity parameters and microbiota abundances in these patients. Secondarily, possible correlations with patients' oral, clinical and habitual characteristics before aSCT should be investigated. |
| Methods | | | |  |
| Study design | 4 | Present key elements of study design early in the paper | 5/6 | First section of Material and methods |
| Setting | 5 | Describe the setting, locations, and relevant dates, including periods of recruitment, exposure, follow-up, and data collection | 5/6 |  |
| Participants | 6 | (*a*) *Cohort study*—Give the eligibility criteria, and the sources and methods of selection of participants. Describe methods of follow-up  *Case-control study*—Give the eligibility criteria, and the sources and methods of case ascertainment and control selection. Give the rationale for the choice of cases and controls  *Cross-sectional study*—Give the eligibility criteria, and the sources and methods of selection of participants | 5/6 |  |
|  |  | (*b*) *Cohort study*—For matched studies, give matching criteria and number of exposed and unexposed  *Case-control study*—For matched studies, give matching criteria and the number of controls per case | N.a. |  |
| Variables | 7 | Clearly define all outcomes, exposures, predictors, potential confounders, and effect modifiers. Give diagnostic criteria, if applicable | 6 |  |
| Data sources/ measurement | 8* | For each variable of interest, give sources of data and details of methods of assessment (measurement). Describe comparability of assessment methods if there is more than one group | 6, 7 |  |
| Bias | 9 | Describe any efforts to address potential sources of bias | 5-7 | “…adhering to the STROBE-Statement for cohort studies”  “Saliva, mucosal biofilm, and stool samples were collected before (T0) and 28 days (±14d) post-allogeneic stem cell transplantation (T1) using standardized and validated techniques”  “The calibration procedure was conducted as long as an interrater reliability of at least 95% regarding the plaque index was achieved” |
| Study size | 10 | Explain how the study size was arrived at | 7 | The sample size for this explorative analysis was determined based on the available resources during the recruitment period. |

Continued on next page

| Quantitative variables | 11 | Explain how quantitative variables were handled in the analyses. If applicable, describe which groupings were chosen and why | 7 | Variables were tested for normal distribution using the Shapiro-Wilk test. Given the non-normal distribution of most continuous variables, median values and 25% and 75% quartiles were calculated. Intergroup differences between patients with and without ulcerations were assessed using the Mann-Whitney-U-Test for continuous variables and Pearson-Chi-square-Test for categorical variables. The significance level (alpha-level) was set at 0.05. |
| --- | --- | --- | --- | --- |
| Statistical methods | 12 | (*a*) Describe all statistical methods, including those used to control for confounding | 7, 8 | See Statistcal methods |
|  |  | (*b*) Describe any methods used to examine subgroups and interactions | 7, 8 | See Statistcal methods |
|  |  | (*c*) Explain how missing data were addressed | NA |  |
|  |  | (*d*) *Cohort study*—If applicable, explain how loss to follow-up was addressed  *Case-control study*—If applicable, explain how matching of cases and controls was addressed  *Cross-sectional study*—If applicable, describe analytical methods taking account of sampling strategy | NA |  |
|  |  | (*e*) Describe any sensitivity analyses | NA |  |
| Results | | | | |
| Participants | 13* | (a) Report numbers of individuals at each stage of study—eg numbers potentially eligible, examined for eligibility, confirmed eligible, included in the study, completing follow-up, and analysed | 8 |  |
|  |  | (b) Give reasons for non-participation at each stage | 8 |  |
|  |  | (c) Consider use of a flow diagram | 9 |  |
| Descriptive data | 14* | (a) Give characteristics of study participants (eg demographic, clinical, social) and information on exposures and potential confounders | 8, 9 |  |
|  |  | (b) Indicate number of participants with missing data for each variable of interest | 8, 9 |  |
|  |  | (c) *Cohort study*—Summarise follow-up time (eg, average and total amount) | 8, 9 |  |
| Outcome data | 15* | *Cohort study*—Report numbers of outcome events or summary measures over time | *8, 9* | *Table 1, Figure 2* |
|  |  | *Case-control study—*Report numbers in each exposure category, or summary measures of exposure | *NA* |  |
|  |  | *Cross-sectional study—*Report numbers of outcome events or summary measures | *NA* |  |
| Main results | 16 | (*a*) Give unadjusted estimates and, if applicable, confounder-adjusted estimates and their precision (eg, 95% confidence interval). Make clear which confounders were adjusted for and why they were included | 8- 16 | Text, tables and figures |
|  |  | (*b*) Report category boundaries when continuous variables were categorized | NA |  |
|  |  | (*c*) If relevant, consider translating estimates of relative risk into absolute risk for a meaningful time period | NA |  |

Continued on next page

| Other analyses | 17 | Report other analyses done—eg analyses of subgroups and interactions, and sensitivity analyses | 8-16 | Text, Tables and Figures throughout the results-section |
| --- | --- | --- | --- | --- |
| Discussion | | | | |
| Key results | 18 | Summarise key results with reference to study objectives | 17 | In this cohort of patients undergoing allogeneic stem cell transplantation, we found a statistically significant decline in α-diversity across all three investigated compartments, though this did not appear to be significantly associated with the development of ulcerations. Moreover, shifts in overall bacterial composition, specifically in beta-diversity, were found in the saliva and stool microbiomes of patients with ulcerations and in the mucosal microbiome of patients without ulcerations. However, in the mucosal samples of patients with ulcerations, the changes of the microbiome structure during a SCT approached the threshold of significance (*p* = 0.055) but still was statistically non-significant. The β-diversity of saliva and mucosal swab samples was significantly influenced by the number of different broad spectrum antibiotics administered during the aSCT period, as well as by gender. Additionally, specific bacterial taxa showed significant changes in abundance before and after aSCT. |
| Limitations | 19 | Discuss limitations of the study, taking into account sources of potential bias or imprecision. Discuss both direction and magnitude of any potential bias | 20, 21 | See the limitations section et the third last section of the discussion |
| Interpretation | 20 | Give a cautious overall interpretation of results considering objectives, limitations, multiplicity of analyses, results from similar studies, and other relevant evidence | 20 | In summary, based on our data and concordant with previous research, it could be cautiously interpreted that a dysbiotic microbiome, enriched with pathogens like *Mycoplasma salvarium*, could be one factor, contributing to the severity of mucositis. |
| Generalisability | 21 | Discuss the generalisability (external validity) of the study results | 20/21 | Included in limitations´ discussion |
| Other information | |  | | |
| Funding | 22 | Give the source of funding and the role of the funders for the present study and, if applicable, for the original study on which the present article is based | 1 | **Funding statement**  The present study was funded by Deutsche Gesellschaft für Präventivzahnmedizin and CP GABA. |

*Give information separately for cases and controls in case-control studies and, if applicable, for exposed and unexposed groups in cohort and cross-sectional studies.

**Note:** An Explanation and Elaboration article discusses each checklist item and gives methodological background and published examples of transparent reporting. The STROBE checklist is best used in conjunction with this article (freely available on the Web sites of PLoS Medicine at http://www.plosmedicine.org/, Annals of Internal Medicine at http://www.annals.org/, and Epidemiology at http://www.epidem.com/). Information on the STROBE Initiative is available at www.strobe-statement.org.
