# Supplementary material for: Exploring the Influence of Oral and Gut Microbiota on Ulcerative Mucositis: A Pilot Cohort Study
Source: Oral Dis. 2025 Jan 6;31(6):1776–88. doi: 10.1111/odi.15246 (PMC12291438; doi:10.1111/odi.15246)
Supplement: Supplementary file 6 — Table S2. Questionnaire participants had to fulfill regarding self‐reported periodontal health. [file ODI-31-1776-s011.docx]

Supplementary Table 2: Questionnaire participants had to fulfil regarding self-reported periodontal health

Do you think you might have gum/dental disease?

1 = Yes

2 = No

3 = I do not know

Overall, how would you rate the health of your teeth and gums?

1 = excellent

2 = very good

3 = good

4 = moderate

5 = poor

6 = I do not know

Have you ever had treatment for gum/ periodontal disease such as "periodontosis/ periodontitis" for example by cleaning under the gums?

1 = Yes

2 = No

3 = I do not know

Have any teeth ever loosened on their own, without previous injury?

1 = Yes

2 = No

3 = I do not know

Have you ever been told by a dentist that you have lost bone around your teeth?

1 = Yes

2 = No

3 = I don't know

In the last three months, have you noticed a tooth that doesn't look right?

1 = Yes

2 = No

3 = I do not know

How often have you flossed or used another tool to clean between your teeth in the past seven days? Number of days_______

How often in the last seven days did you use mouthwash or other dental rinse that you use to treat dental disease or problems? Number of days_____
