# Supplementary material for: Exploring the Influence of Oral and Gut Microbiota on Ulcerative Mucositis: A Pilot Cohort Study
Source: Oral Dis. 2025 Jan 6;31(6):1776–88. doi: 10.1111/odi.15246 (PMC12291438; doi:10.1111/odi.15246)
Supplement: Supplementary file 7 — Table S3. Number of patients with Conditioning drugs, Total Body Irradiation (TBI) and other supportive medications. [file ODI-31-1776-s001.docx]

Supplementary table 3: Number of patients with Contidoning drugs, Total Body Irradiation (TBI) and other supportive medications

|  |  | all | u- | u+ |
| --- | --- | --- | --- | --- |
| Conditioning agents | Fludrabin | 23 | 14 | 9 |
|  | Melphalan | 3 | 1 | 2 |
|  | Busulfan | 4 | 1 | 3 |
|  | Treosulfan | 7 | 6 | 1 |
|  | Cyclophosphamid | 2 | 2 | 0 |
|  | Clofarabin | 1 | 0 | 1 |
|  | Cytorabin | 2 | 0 | 2 |
|  | Thiotepa | 1 | 1 | 0 |
| Chemotherapy | Daunorubicin | 1 | 0 | 1 |
|  | HAM | 3 | 1 | 2 |
| TBI | | 10 | 7 | 3 |
|  | Dosage 2 Gy | 1 | 1 | 0 |
|  | Dosage 6 Gy | 3 | 1 | 2 |
|  | Dosage 8 Gy | 6 | 5 | 1 |
| Immunosuppressants | Methotrexat | 16 | 9 | 7 |
|  | Cyclosporin A | 23 | 14 | 9 |
|  | Anti-Thymocyte Globulin | 22 | 12 | 10 |
|  | Cellcept | 2 | 2 | 0 |
|  | Mycophenola | 1 | 1 | 0 |
|  | Sirolimus | 1 | 1 | 0 |
|  | Tacrolimus | 2 | 0 | 2 |
| Glucocorticoids | Dexamethson | 5 | 4 | 1 |
|  | other Glukocortikoid | 16 | 9 | 7 |
|  | Leucoverin | 17 | 10 | 7 |
|  | Insulin | 3 | 1 | 2 |
| Borad-spectrum antibiotics | Tazobactam | 6 | 5 | 1 |
|  | Cotrim forte | 25 | 15 | 10 |
|  | Ciprofloxacin | 4 | 2 | 2 |
|  | Amoxicillin/Clavulanic acid | 5 | 3 | 2 |
|  | Amikacin | 1 | 1 | 0 |
|  | Meronem | 3 | 3 | 0 |
|  | Rifaximin | 13 | 7 | 6 |
|  | Ceftazidime/Avibactam | 1 | 1 | 0 |
|  | Antimycotic drugs | 25 | 15 | 10 |
|  | Vantiviral drugs | 25 | 15 | 10 |
|  | Vitamin D | 14 | 8 | 6 |

*Note* HAM = High-dose Cytarabine + Mitoxantrone
