# Supplementary material for: Exploring the Influence of Oral and Gut Microbiota on Ulcerative Mucositis: A Pilot Cohort Study
Source: Oral Dis. 2025 Jan 6;31(6):1776–88. doi: 10.1111/odi.15246 (PMC12291438; doi:10.1111/odi.15246)
Supplement: Supplementary file 8 — Table S4. Reads counts for the mock community with 3 replicates. [file ODI-31-1776-s009.docx]

Supplementary table 4: Reads counts for the mock community with 3 replicates.

| Genus | Mock_rep1 | Mock_rep2 | Mock_rep3 |
| --- | --- | --- | --- |
| Limosilactobacillus | 1409 | 1973 | 1522 |
| Bacillus | 891 | 1180 | 914 |
| Staphylococcus | 828 | 1069 | 851 |
| Salmonella | 703 | 1021 | 772 |
| Listeria | 683 | 855 | 667 |
| Escherichia-Shigella | 615 | 850 | 674 |
| Enterococcus | 350 | 450 | 409 |
| Pseudomonas | 349 | 483 | 424 |
