# Supplementary material for: Exploring the Influence of Oral and Gut Microbiota on Ulcerative Mucositis: A Pilot Cohort Study
Source: Oral Dis. 2025 Jan 6;31(6):1776–88. doi: 10.1111/odi.15246 (PMC12291438; doi:10.1111/odi.15246)
Supplement: Supplementary file 9 — Appendix S1. Fragebögen Teil 1–3. [file ODI-31-1776-s012.docx]

**Fragebögen für Studienteilnehmer(innen): Teil 1-3**

Bitte Teil 1-3 am **Tag vor oder am ersten Tag des Aufenthalts auf Station** so weit wie möglich ausfüllen.

Die Fragen von Teil 1-3 beziehen sich auf die Zeit **vor** dem stationären Aufenthalt/**vor** Beginn der Stammzelltransplantation.

Datum: ______________

Name, Vorname: ___________________________

**Teil 1. FRAGEN ZUR LEBENSSITUATION**:

(Schul-)Bildung: 🞎 kein Schulabschluss

🞎 Volksschulabschluss/Abschluss 8. Klasse/Hauptschulabschluss

🞎 Mittlere Reife /Abschluss 10. Klasse/anderer Schulabschluss

🞎 Fachhochschulreife/ Abitur

🞎 keine Berufsausbildung

🞎 abgeschlossener Berufsausbildung

🞎 Fachhochschulabschluss

🞎 Hochschulabschluss

Beruf: _______________________ 🞎vollzeit 🞎teilzeit 🞎arbeitslos gemeldet

🞎angestellt 🞎selbstständig 🞎Rentner 🞎Hausfrau/Hausmann 🞎nicht (mehr) arbeitsfähig

(Haushalts-)Einkommen: ____________________ monatlich (netto, nach Abzügen)

Familienstatus: 🞎ledig 🞎verheiratet 🞎geschieden 🞎verwitwet

Anzahl von Kindern: _________

**Teil 2. FRAGEN ZUR MUNDGESUNDHEIT:**

Mundhygiene und Ernährung:

Wie häufig besuchen Sie den Zahnarzt?_________________________/ Jahr

Wie oft reinigen Sie sich die Zähne?_________________/ Tag

Wie lange dauert der Putzvorgang durchschnittlich?___________min.

Welche Zahnbürste verwenden Sie? 🞎Handzahnbürste 🞎elektrische Zahnbürste 🞎beides

Wie oft tauschen Sie die Zahnbürste? ______________/Jahr

Welche Zahnpasta verwenden Sie? 🞎mit Fluorid 🞎ohne Fluorid

Präparat angeben ___________________________________________________________

Verwenden Sie weitere Hilfsmittel zur Mundhygiene? 🞎 ja 🞎nein

Wenn ja, welche? 🞎Zahnseide 🞎Zahnzwischenraumbürstchen 🞎Andere:_________________

Wie oft? _______________/Tag _______________/Woche

Verwenden Sie Chlorhexidinpräparate? 🞎 ja 🞎nein

Verwenden Sie Mundspüllösungen (z.B. mit ätherische Ölen /Listerine, CPC, Triclosan oder Fluoriden)?

🞎 ja 🞎 nein

Wenn ja, welche? (Präparat angeben)________________________________

Wie häufig essen Sie zuckerhaltige/gesüßte Produkte? __________/ Tag

Wie häufig trinken Sie zuckerhaltige/ gesüßte Getränke? __________/ Tag

Wie häufig essen oder trinken Sie Obst, saure Getränke? __________/ Tag ______/Woche

oder Milch-Produkte __________/ Tag ______/Woche

Verwenden Sie Produkte mit Calcium? 🞎 ja 🞎nein

Wenn ja, wie oft? ____/ Tag ___/Woche

Verwenden Sie Probiotika? 🞎ja 🞎nein

Wenn ja, wie oft? ______/ Tag ______/Woche Welche?_______________

Bevorzugen Sie überwiegend Vollwert-Kost? 🞎 ja 🞎 nein

Sind Sie Vegetarier? 🞎 ja 🞎 nein

Wie häufig kauen Sie Kaugummi? __________/ Tag

Verwenden Sie Produkte mit Xylit? 🞎ja 🞎nein

Wenn ja, wie oft? ____/ Tag ____/Woche

Zahnfleisch und Zahnhalteapparat:

Denken Sie, dass Sie eine Zahnfleisch-/Zahnhalteapparat-Erkrankung haben könnten?

🞎ja 🞎nein 🞎ich weiß nicht

Wie beurteilen Sie insgesamt die Gesundheit Ihrer Zähne und Ihres Zahnfleisches?

🞎ausgezeichnet 🞎sehr gut 🞎gut 🞎mäßig 🞎schlecht 🞎ich weiß nicht

Hatten Sie jemals eine Behandlung von Zahnfleisch-/Zahnhalteapparat- Erkrankung wie „Parodontose/ Parodontitis“ zum Beispiel durch Reinigung unter dem Zahnfleisch?

🞎ja 🞎nein 🞎 ich weiß nicht

Haben sich schon einmal von selbst, ohne vorangegangene Verletzungen, irgendwelche Zähne gelockert?

🞎ja 🞎nein 🞎ich weiß nicht

Wurden Sie jemals von einem Zahnarzt darauf hingewiesen, dass Sie Knochen um Ihre Zähne herum verloren haben?

🞎ja 🞎nein 🞎ich weiß nicht

Haben Sie in den letzten drei Monaten einen Zahn bemerkt, der nicht richtig aussieht?

🞎ja 🞎nein 🞎ich weiß nicht

Abgesehen davon, dass Sie Ihre Zähne mit einer Zahnbürste geputzt haben, wie oft haben Sie in den letzten sieben Tagen Zahnseide oder ein anderes Hilfsmittel verwendet, um zwischen Ihren Zähnen zu reinigen?

an _________ Tagen

Abgesehen davon, dass Sie Ihre Zähne mit einer Zahnbürste geputzt haben, wie oft haben Sie in den letzten sieben Tagen Mundwasser oder andere Zahnspülmittel verwendet, die Sie zur Behandlung von Zahnerkrankungen oder Zahnproblemen verwenden?

an _________ Tagen

Alkoholkonsum: 🞎 keiner/gering (einmal oder weniger pro Monat)

🞎moderat (einige Male pro Monat)

🞎häufig (wöchentlich)

🞎sehr häufig (täglich)

Zigarettenkonsum:

Sind/Waren Sie Raucher? 🞎 ja 🞎nein

Wenn ja/ehemals: ___________________ Zigaretten pro Tag

Seit/bis wann: ___________________

**Teil 3. FRAGEN ZUR LEBENSQUALITÄT:**

Bitte die nachfolgend beiliegenden Fragebögen QLQ-C30 und QLQ-OH15 in Bezug auf die letzte Woche vor Ihrem stationären Aufenthalt ausfüllen.
