# Supplementary material for: Exploring the Influence of Oral and Gut Microbiota on Ulcerative Mucositis: A Pilot Cohort Study
Source: Oral Dis. 2025 Jan 6;31(6):1776–88. doi: 10.1111/odi.15246 (PMC12291438; doi:10.1111/odi.15246)
Supplement: Supplementary file 11 — Appendix S3. OH15 German. [file ODI-31-1776-s005.pdf]

# EORTC QLQ – OH15

Patienten berichten manchmal die folgenden Symptome oder Probleme. Bitte geben Sie an, in welchem Ausmaß Sie diese Symptome oder Probleme während der letzten Woche erlebt haben. Markieren Sie bitte die Zahl, die am besten auf Sie zutrifft.

| Während der letzten Woche: | Überhaup nicht | Wenig | Mäßig | Sehr |
|---|---|---|---|---|
| 31. Hatten Sie Schmerzen am Zahnfleisch? | 1 | 2 | 3 | 4 |
| 32. Hatten Sie Probleme mit Zahnfleischbluten? | 1 | 2 | 3 | 4 |
| 33. Hatten Sie wunde Lippen? | 1 | 2 | 3 | 4 |
| 34. Hatten Sie Probleme mit den Zähnen? | 1 | 2 | 3 | 4 |
| 35. Hatten Sie Schmerzen im Mund? | 1 | 2 | 3 | 4 |
| 36. Hatten Sie wunde Stellen in den Mundwinkeln? | 1 | 2 | 3 | 4 |
| 37. Hatten Sie einen trockenen Mund? | 1 | 2 | 3 | 4 |
| 38. Hatten Sie klebrigen Speichel? | 1 | 2 | 3 | 4 |
| 39. Hat Ihr Mund empfindlich auf Essen oder Trinken reagiert? | 1 | 2 | 3 | 4 |
| 40. War Ihr Geschmacksempfinden beim Essen oder Trinken verändert? | 1 | 2 | 3 | 4 |
| 41. Hatten Sie Probleme beim Essen fester Nahrung? | 1 | 2 | 3 | 4 |

| Während der letzten Woche: | Ja | Nein |
|---|---|---|
| 42. Haben Sie eine Zahnprothese getragen? | 1 | 2 |

| Bitte beantworten Sie die Frage 43 nur, wenn Sie eine Zahnprothese getragen haben: | Überhaup nicht | Wenig | Mäßig | Sehr |
|---|---|---|---|---|
| 43. Hatten Sie Probleme mit einer schlecht sitzenden Zahnprothese? | 1 | 2 | 3 | 4 |

| Während Ihrer derzeitigen Erkrankung oder Behandlung, nicht nur während der letzten Woche: | Ja | Nein |
|---|---|---|
| 44. Haben Sie Informationen über mögliche Zahn- oder Mundprobleme erhalten? | 1 | 2 |

| Bitte beantworten Sie die Frage 45 nur, wenn Sie Informationen erhalten haben: | Überhaup nicht | Wenig | Mäßig | Sehr |
|---|---|---|---|---|
| 45. Sind Sie mit der Menge der erhaltenen Informationen über mögliche Zahn- oder Mundprobleme zufrieden gewesen? | 1 | 2 | 3 | 4 |

© Copyright 2016 EORTC Quality of Life Group. Alle Rechte vorbehalten. Phase IV module.
