# Supplementary material for: Exploring the Influence of Oral and Gut Microbiota on Ulcerative Mucositis: A Pilot Cohort Study
Source: Oral Dis. 2025 Jan 6;31(6):1776–88. doi: 10.1111/odi.15246 (PMC12291438; doi:10.1111/odi.15246)
Supplement: Supplementary file 12 — Appendix S4. Fragebögen Teil 4. [file ODI-31-1776-s003.docx]

**Fragebögen für Studienteilnehmer(innen): Teil 4**

Bitte Teil 4 **während der Stammzelltransplantation** ausfüllen.

Datum des Transplantationsbeginns: ______________

Name, Vorname: ___________________________

**Teil 4. FRAGEN ZUR ERFASSUNG DES GRADS DER MUNDSCHLEIMHAUT ENTZÜNDUNG**

Bitte am ersten Tag nach der Stammzelltransplantation und darauffolgende alle 3 Tage die zutreffenden Antworten auf den nachfolgenden Seiten ankreuzen und das Datum vermerken. Bitte auch Abweichungen der Zeitspanne notieren um uns bei der Auswertung zu helfen.

**HERZLICHEN DANK IM VORAUS!**

**Teil 4. FRAGEN ZUR ERFASSUNG DES GRADS DER MUNDSCHLEIMHAUT ENTZÜNDUNG**

**Tag 1** nach Transplantation: Bitte die zutreffenden Antworten ankreuzen und das Datum vermerken.

Datum: _______

**Teil 4. FRAGEN ZUR ERFASSUNG DES GRADS DER MUNDSCHLEIMHAUT ENTZÜNDUNG**

**Tag 4** nach Transplantation: Bitte die zutreffenden Antworten ankreuzen und das Datum vermerken.

Datum: _______

**Teil 4. FRAGEN ZUR ERFASSUNG DES GRADS DER MUNDSCHLEIMHAUT ENTZÜNDUNG**

**Tag 8** nach Transplantation: Bitte die zutreffenden Antworten ankreuzen und das Datum vermerken.

Datum: _______

**Teil 4. FRAGEN ZUR ERFASSUNG DES GRADS DER MUNDSCHLEIMHAUT ENTZÜNDUNG**

**Tag 12**  nach Transplantation: Bitte die zutreffenden Antworten ankreuzen und das Datum vermerken.

Datum: _______
